# Discrepant Expression of Cytokines in Inflammation- and Age-Related Cataract Patients

**DOI:** 10.1371/journal.pone.0109647

**Published:** 2014-10-10

**Authors:** Wan Chen, Haotian Lin, Xiaojian Zhong, Zhaochuan Liu, Yu Geng, Chufang Xie, Weirong Chen

**Affiliations:** State Key Laboratory of Ophthalmology, Zhongshan Ophthalmic Center, Sun Yat-sen University, Guangzhou, Guangdong, China; University of Thessaly, Faculty of Medicine, Greece

## Abstract

**Purpose:**

Inflammatory cataracts secondary to Behcet's disease (BD) or Vogt-Koyanagi-Harada disease (VKH) are thought to result from a pathological dysregulation of cytokines that is different from that of age-related (AR) cataracts. However, little is known about the function of cytokines in the development of inflammatory cataracts. The purpose of this study was to identify possible differences in cytokine expression in inflammation- and age-related cataract patients.

**Methods:**

Analysis techniques involving the concomitant use of a cocktail of antibody-coated non-magnetic beads were used to determine the cytokine expression profiles of BD, VKH and AR cataract patients. Furthermore, anterior chamber aqueous flares and inflammatory cells were quantitatively measured with a laser flare cell meter (LFCM).

**Results:**

The expressions of interleukin-2 (IL-2), IL-4, IL-6, IL-10, IL-17A, and interferon-γ (IFN-γ) were analyzed in aqueous humor (AqH), phytohemagglutinin (PHA)-stimulated and non-PHA-stimulated cultures of peripheral blood mononuclear cells (PBMCs) from the three types of cataract patients. IL-6 and IFN-γ were identified above the detection limits, but, among the BD and VKH cataract patients, only the levels of IL-6 were significantly higher in both the AqH and PBMC non-PHA cultures compared with the levels observed in the AR cataract patients. In contrast, IFN-γ was significantly elevated in the AqH of the BD cataract patients compared with the VKH and AR cataract patients. In the PHA-stimulated PBMC cultures, IL-2, IFN-γ, IL-6, and IL-17A were significantly increased, and the IL-6 level was significantly higher in the VKH patients than in the BD and AR cataract patients. The correlation analyses of the cytokines and inflammation indexes of the AqH obtained with the LFCM revealed that only IL-6 was significantly correlated with the inflammation index.

**Conclusion:**

Distinct expression profiles of cytokines and the correlations of these profiles with in vivo inflammatory indexes for inflammatory and AR cataract patients were identified.

## Introduction

Cataracts secondary to uveitis, such as those that occur in Behcet’s disease (BD) and Vogt-Koyanagi-Harada disease (VKH), challenge ophthalmologists in many aspects, including a miotic pupil, iris atrophy, posterior synechiae, pupillary membrane, operation time, perioperative medication, macular edema, severe postoperative inflammation and band keratopathy. The pathogenesis of this type of cataract is thought to be inflammation-related, in contrast to the pathogenesis of age-related (AR) cataracts. However, the expression patterns and function of cytokines during the development of inflammatory cataracts remain unknown.

Cytokines are crucially involved in the regulation of the normal human immune response. Dysregulated cytokine expression participates in the pathogenesis of autoimmune diseases, including BD and VKH. [1]–[3] Investigations of cytokine expression have advanced our understanding of the pathogeneses of various diseases. Previous studies have provided increasing support for the hypothesis that specific CD4^+^T helper (Th) cell-mediated immune responses play a central role in the pathogenesis of uveitis. [4] CD4^+^ T cells are crucial for immunoregulation because they orchestrate the function of other immune cell types. CD4^+^ T cells can be sub-classified into groups, which include Th1, Th2, and Th17 cells. Th1 cells, which predominantly secrete IFN-γ, may play a pathogenic role in organ-specific autoimmune and other chronic inflammatory disorders. [5], [6] Th2 cells, which primarily produce IL-4, IL-5, and IL-13, exert a strong inhibitory effect on Th1 differentiation and are responsible for the pathogenesis of allergic diseases. [7] Previous studies have suggested that both BD and VKH are predominantly related to the Th1 immune response, and patients with these diseases have increased levels of Th1-associated cytokines, such as interferon-gamma (IFN-γ), interleukin-12 (IL-12), and TNF-a. [8]–[11] Recently, several investigators have detected increased levels of IL-17 in active BD and VKH patients relative to patients in inactive stages and control healthy subjects. [11]–[14] Based on these findings, Th17 cells, in addition to Th1 cells, might be involved in the pathogeneses of these two subtypes of uveitis. Therefore, we inferred that there might be differences in the expression levels of IL-2, IL-4, IL-6, IL-10, IL-17A, and IFN-γ between inflammatory cataracts that are secondary to BD and VKH and AR cataracts. Long-standing ocular inflammation is a characteristic of uveitis. Previous studies have shown that aqueous flare can be detected in uveitis patients with long-standing inflammation even when that inflammation has been quiet for 3 months or longer. [15] Therefore, there is also likely to be differences in the aqueous flares and inflammatory cells of patients with BD and VKH cataracts relative to those with AR cataracts.

In this study, we concomitantly used a cocktail of antibody-coated non-magnetic beads to determine the cytokine expression profiles of BD, VKH and AR cataract patients, and we used a laser flare cell photometer (LFCM) to quantitatively measure the anterior chamber aqueous flares and inflammatory cells of these patients.

## Methods

### Patients

Nine patients (8 males and 1 female) with cortical cataracts secondary to BD (age 41.11±7.22 years) and ten VKH cortical cataract patients (4 males and 6 females, age 47.10±9.78 years) who required cataract surgery between January, 2010 and June, 2011 at the Cataract Treatment Department of Zhongshan Ophthalmic Center were included in the study. BD and VKH were diagnosed based on the International Study Group for Behcet’s Disease Criteria [16] and the Revised International Diagnostic Criteria for VKH [17], respectively. There was no significant difference in the duration of disease between the VKH and BD patients (P = 0.895). All patients had received low dose prednisone (<20 mg/d) for at least 3 months to ensure that their uveitis was inactive prior to inclusion in this study. Ten age-matched AR cortical cataract patients (4 males and 6 females, age 49.90±10.59 years) with no prior history of uveitis, glaucoma, ocular trauma, rheumatoid arthritis, diabetes or other eye or systematic diseases were also included at the same time period. The three types of cortical cataract patients were matched for age and sex (P>0.05). This study was performed in accordance with the Declaration of Helsinki and with the approval of the Sun Yat-sen University-Zhongshan Ophthalmic Center-Institutional Review Board (SYSU-ZOC-IRB). Written informed consent was obtained from all patients.

### Measurement of Preoperative Anterior Chamber Inflammation

Quantitative measurements of anterior chamber aqueous flare and inflammatory cells were obtained preoperatively for the BD, VKH and AR cataract patients with a FC-2000 LFCM (Kowa, Tokyo, Japan) as described in a previous study [18]. Three individual measurements from each eye were averaged, and measurements that were affected by artifacts were discarded. Flare and cell readings are expressed as photon counts per millisecond (pc/ms) and cells/0.5 mm^3^, respectively.

### Analyses of the Cytokine Expression Profiles

All 29 included patients agreed to provide samples of peripheral blood (10 ml) and aqueous humor (100–200 µl) prior to their cataract surgeries. Peripheral blood mononuclear cells (PBMCs) were prepared from 10 ml of fresh heparinized blood via Ficoll-Hypaque density-gradient centrifugation. To study the production of the related cytokines, the PBMCs were cultured either with or without phytohemagglutinin (PHA) stimulation at a density of 2×10^6^ cells/ml for 72 hours. Then, the supernatants were harvested and stored at −70°C until the cytokine analyses. Aqueous humor (100–200 µl) was aspirated from each patient via limbic paracentesis using a 27-gauge needle attached to a tuberculin syringe following the topical application of a local anesthetic, oxybuprocaine hydro-chloride (0.4%, Benoxinate, Chauvin Pharmaceuticals Ltd., Kingston, United Kingdom). The procedure was performed under a surgical microscope before phacoemulsification began. The samples were snap frozen and maintained at −70°C until use. Aqueous humor samples were obtained from all patients with uveitis at the beginning of the surgeries.

The cytokine expression profiles in the supernatants of the PBMC cultures and the aqueous humor samples were determined using a cocktail of antibody-coated non-magnetic beads that enabled concomitant measurements of the levels of IFN-γ, IL-2, IL-4, IL-6, IL-10, and IL-17A (Bio-plex pro-cytokine assay, BIORAD, Hercules, CA, USA). This technology allowed multiple analyses to be conducted on a single 50-μl sample. The analysis was performed following the manufacturer's instructions, and the results were generated using the Bio-Plex 200 system and software.

### Statistical Analyses

The data were analyzed using the Statistical Package for Social Sciences (SPSS Version 16.0 for Windows). All data are expressed as the mean ± SD. One-way ANOVA and post-ANOVA pairwise comparisons of the means were conducted using the Kruskal-Wallis test to analyze the differences among the groups. The Mann-Whitney test was also used where indicated by the results of tests of the assumptions of normality and the homogeneity of the variance. Linear regression was used to assess correlations between pairs of variables. For all tests, P≤0.05 was considered statistically significant.

## Results

### Anterior Chamber Inflammation

The preoperative flare values of the VKH and BD groups were 18.21±4.80 and 21.72±3.24 pc/ms, respectively, and both of these values were significantly greater than those of the AR group (P = 0.001 and P<0.001, respectively). However, no significant difference was found between the two uveitis groups (P = 0.123, Table 1). The inflammatory cell counts in the VKH, BD, and AR groups were 6.53±3.54, 5.80±3.21, and 4.03±2.73 cells/0.5 mm^3^, respectively. No significant differences were found among these groups (F = 1.837, P = 0.179, Table 1).

**Table 1:** Anterior chamber aqueous flare and inflammatory cells counts of the three groups.

Inflammation parameters	VKH Group	BD Group	AR Group	F	P
Flare values (pc/ms)	18.21±4.80*	21.72±3.24*	5.80±3.17	54.883	0.000
Cell count (cells/0.5 mm^3^)	6.53±3.54	5.80±3.21	4.03±2.73	1.837	0.179

**Footnotes:** *P<0.05 versus the AR samples.

### Cytokine Concentrations in the Supernatants of the PBMC Cultures

**Figure 1:**
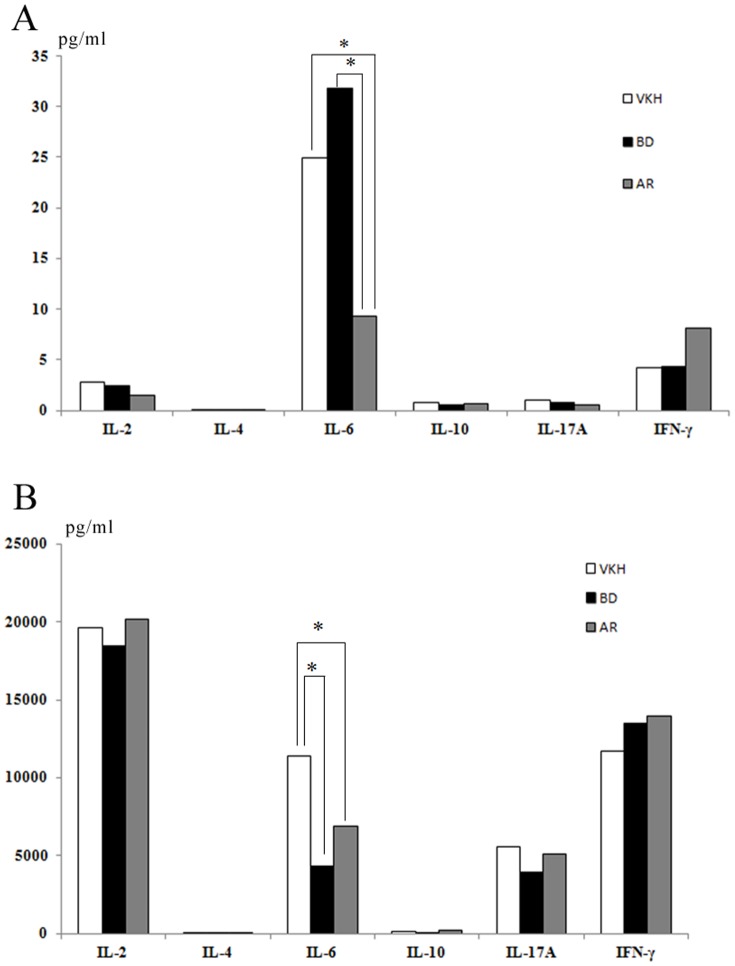
Mean IL-2, IL-4, IL-6, IL-10, IL-17A and IFN-γ levels in the PBMC samples from the VKH and BD patients and the ARs (pg/ml). Panel A, without PHA stimulation; Panel B, with PHA stimulation. *Statistically significant at the 5% level.

The IL-2, IL-4, IL-10 and IL-17A contents were below the detection levels in the non-PHA-stimulated PBMC samples of the patients and the AR group. IFN-γ was detected, but no significant differences were found among the three groups (P = 0.583, Figure 1). However, we observed that IL-6 was significantly elevated in both the BD and VKH patients compared with the AR cataract patients (P = 0.017 and P = 0.004, respectively, Figure 1-A).

IL-2, IL-6, IL-17A, and IFN-γ were significantly increased in all of the PBMC samples following PHA stimulation, while IL-4 and IL-10 remained at relatively low levels (Figure 1-B). However, no significant differences in the mean levels of the above-mentioned cytokines were observed among the groups (Figure 1). Moreover, IL-6 was significantly increased in the VKH patients compared with the BD patients and the AR cataract patients (P = 0.000 and P = 0.013, respectively, Figure 1-B).

### Cytokine Levels in the Aqueous Humor

The mean cytokine levels among the three groups were compared using the Kruskal-Wallis test, and the results are shown in Table 2. IL-6 and IFN-γ were detected in all aqueous humor samples from the patients with uveitis and the AR group, but the other cytokines were below the detection levels. The IL-6 cytokine levels were significantly higher in the aqueous humors of the VKH and BD patients compared with the AR cataract patients (P = 0.034 and P = 0.003, respectively), and the difference between the two disease groups was not significant (P = 0.921). Moreover, the IFN-γ levels in the aqueous humor samples of the BD patients were significantly higher than those of both the VKH and AR groups (P = 0.001 and P = 0.014, respectively).

**Table 2:** Comparisons of the mean cytokine levels in the aqueous humor samples among the three groups via ANOVA.

Cytokine	Inflammatory Groups		AR Group	F	P
	VKH	BD			
IL-2	<1.16	<1.16	<1.16		
IL-4	<0.05	<0.05	<0.05		
IL-6	22.96±6.68*	28.34±17.8*	9.31±6.10	7.373	0.003
IL-10	<0.16	<0.16	<0.16		
IL-17A	<0.46	<0.46	<0.46		
IFN-γ	2.21±0.73	10.57±4.90*	1.83±1.29	53.940	0.000

**Footnotes:** *P <0.05 versus the AR samples.

### Correlations Between Cytokines Levels and Anterior Chamber Inflammation

**Figure 2:**
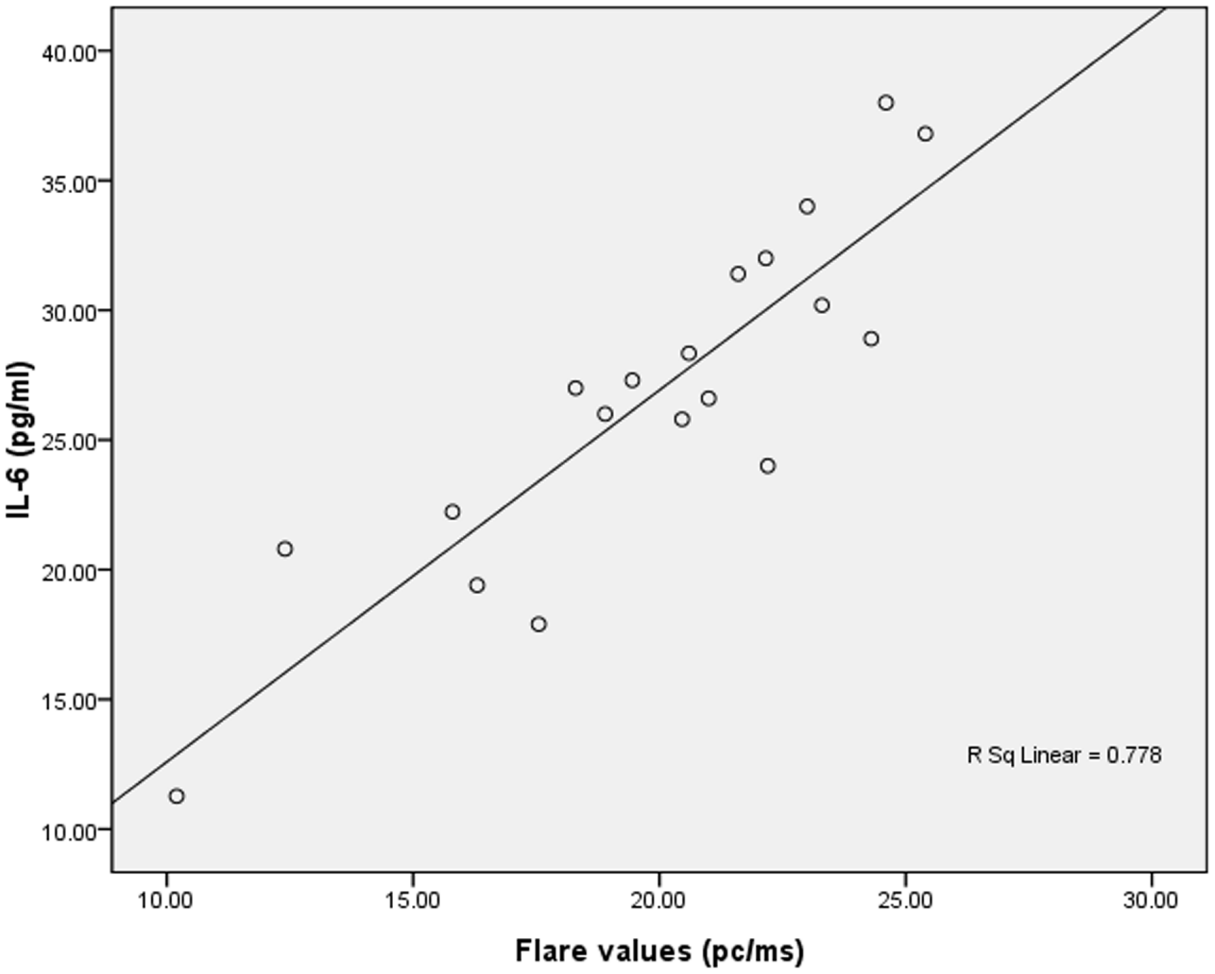
Correlation between the IL-6 levels in the AqH and the preoperative flare values in the VKH and BD patients.

The IL-6 levels in the AqH samples of the VKH and BD patients were positively correlated with the preoperative flare values (r = 0.778 and P = 0.000, respectively, Figure 2). However, we found no correlation between the IL-6 level in the PBMCs and the flare values in the uveitis patients (P  = 0.297 and P = 0.599, respectively). Furthermore, there were no correlations between IL-6 levels and cell counts (P  = 0.250 and P = 0.385, respectively). Although the IFN-γ levels in the AqH samples from the BD patients were significantly higher than the levels observed in the samples from both the VKH and AR groups, we found no correlations between the IFN-γ levels in the AqH and the anterior chamber Flare values (r = 0.481, P = 0.190) and cell counts (r = 0.065, P = 0.790) of the BD patients.

## Discussion

Until now, little was known about the function of cytokines in the development of inflammatory cataracts. Cytokines are crucial in the regulation of normal human immune responses and related diseases. Based on a systematic review of previous studies, we have summarized the expressions of cytokines in patients with VKH and BD in Table 3. We inferred that the expressions of IL-2, IL-4, IL-6, IL-10, IL-17A, and IFN-γ were the most likely to differ between inflammatory cataracts that are secondary to BD and VKH and AR cataracts. Therefore, we concomitantly used a cocktail of antibody-coated non-magnetic beads to determine the expression profiles of these cytokines in BD, VKH and AR cataract patients. The expression levels of IL-2, IL-4, IL-6, IL-10, IL-17A, and IFN-γ were analyzed in the AqH, and PHA-stimulation and non-PHA-stimulated cultures of PBMCs from these three types of cataract patients. In addition, we used LFCM to quantitatively measure the anterior chamber aqueous flares and inflammatory cell levels of these patients to identify any possible differences among the groups of patients and any relationships between the cytokine levels and the clinical inflammation indexes.

**Table 3:** The expression levels of cytokines in the peripheral blood and AqH samples of patients with VKH or BD, as reported in previous studies.

Study (Year)	Patients (n)	Origin	Studied cytokines	Stage of disease	Samples	Main results
Sunao Sugita [19] (2012)	BD (10)	Japan	IL-2, IL-4, IL-6, IL-10, TNF-a, IFN-γ	active (6), inactive (4)	AqH	IFN-γ*, IL-2*, TNF-a*, IL-6*, IL-17*
					supernatants of T-cell cultures	IFN-γ*, TNF-a*, IL- 6*, IL-17*
Ahmed M. Abu El-Asrar [20] (2011)	BD (16), VKH (16)	Saudi Arabia	IL-15, IL-17, IFN-γ, TNF-α and IL-10	≥3 cells in the AC	AqH of BD patients	IFN-γ*, IL-15*, IL-17*, TNF-α*, IL-10*
					AqH of VKH patients	IFN-γ*, IL-10*, IL-17*, TNF-α*, IL-15*
A. G. Commodaro [21] (2010)	VKH (21)	Brazil	IFN-γ, IL-10, TGF-β	active (13), inactive (8)	supernatants of PBMC cultures with PHA stimulation	IFN-γ*, IL-10†, TGF-β^†^
B Li [10] (2005)	VKH (16)	China	IFN-γ, IL-2, IL-4	active (16), inactive (16)	supernatants of PBMC cultures with PHA stimulation and	IFN-γ*, IFN-γ^†^
					spontaneous secretion of PBMCs	
N AKdeniz [22] (2004)	BD (27)	Turkey	IL-2, IL-6, TNF-a	Active stage	Serum	IL-2*, IL-6*, TNF-a*
Midori Misumi [23] (2003)	BD (18)	Japan	TN F-a, IFN-γ, IL-12, IL-4	active (6), inactive (4)	supernatants of PBMC cultures with PHA stimulation	IFN-γ*, IL-4*, IFN-γ^†^
					spontaneous secretion of PBMCs	TNF-a*, IL-12*, IFN-γ*,IFN-γ^†^
Cem Evereklioglu [24] (2002)	BD (37)	Turkey	TNF-a, IL-1β, sIL-2R, IL-6, IL-8	active (17), inactive (20)	Serum	TNF-a*, sIL-2R*, IL-6*, IL-8*, IL-6^†^

**Footnotes:** *Cytokines elevated in the active stage of the disease; ^†^cytokines elevated in the inactive stage of the disease.

In our study, IL-6 was significantly increased in the AqH and the secretions of the PBMCs of both the BD and VKH patients compared with the AR cataract patients, whereas no statistically significant difference was observed between the BD and VKH patients. Previous studies have reported increased levels of IL-6 in the AqH, supernatants of PBMC cultures, the sera of active BD patients, and the AqH and vitreous humor of active VKH patients. [19], [22], [24], [25] Similar to the result of our study, Cem Evereklioglu et al. [24] reported that even inactive patients with BD exhibit significantly higher IL-6 levels compared with AR subjects. IL-6 is a critical mediator of the induction of inflammation and is an essential factor in the differentiation of Th17s. However, there were no signs of acute inflammation in the BD or VKH cataract patients; the inflammatory cells counts of these patients were similar to those of the AR group (F = 1.837, P = 0.179). Therefore, the presence of IL-6 in the AqH samples of these patients could not have merely been the consequence of an acute inflammatory process. Interestingly, we found that the preoperative aqueous flare values were positively correlated with the IL-6 levels in the AqH of the VKH and BD patients but not with the levels in the secretions of the PBMCs; these findings indicate that the elevated IL-6 level in the AqH, rather than some systemic effect, was correlated with a long-term impairment of the blood-aqueous barrier function.

The role of IFN-γ, which is the main signature cytokine of the Th1 lineage, was intensively studied in EAU models in the 1990s. [26] Moreover, the Th1 subset has been thought of as a major pathogenic effector T cell subset since those studies. Most of the previous studies reported increased levels of IFN-γ in the ocular fluids, supernatants of PBMC cultures and sera of active VKH and BD patients. [10], [19]–[21], [23] In the present study, the aqueous IFN-γ level was significantly higher in the patients with BD than in the patients with VKH. This result is consistent with those of Abu El-Asrar et al., [20] who reported that the levels of IFN-γ in the aqueous humor are significantly higher in patients with BD than in patients with VKH and patients with HLA-B27-associated uveitis. Therefore, it is tempting to speculate that the Th1-type immune responses are more potent in patients with BD than in patients with VKH. In this study, we found no significant correlation between IFN-γ levels in the AqH and the flare values in BD patients (r = 0.481, P = 0.190), and this finding is indicative of a weak correlation between the IFN-γ level and the impairment of the blood-aqueous barrier function. However, this finding may be due to the limited number of patients recruited for our study. Another interesting finding is that the IL-17A expression levels were below the detection level in the AqH and PBMC samples from the uveitis patients and AR cataract patients who were not subjected to PHA stimulation. With PHA stimulation, the IL-17A levels were significantly elevated in all of the PBMC samples, and no significant differences among the groups were found. Previous studies have reported high IL17 levels in the peripheral blood and even in the AqH of patients with active VKH or BD but not in these fluids from patients in the inactive phase. [12], [19], [20], [27] These results indicate that IL-17 contributes to the active proinflammatory pattern that is characteristic of inflammatory diseases of patients with active VKH or BD.

The IL-10 expression patterns of VKH and BD patients vary across different studies. Abu El-Asrar [20] reported increased levels of IL-10 in the AqH of active VKH and BD patients. However, Sunao Sugita [19] failed to detect IL-10 in the AqH of active VKH or BD patients. Notably, A. G. Commodaro [21]found high expression levels of IL-10 in the supernatants of PBMC cultures that were stimulated with PHA from inactive VKH patients. The main biological functions of IL-10 seem to be the limitation and termination of inflammatory responses and the regulation of the differentiation and proliferation of several immune cells, such as T cells, B cells, natural killer cells, antigen-presenting cells, and granulocytes. Immunoregulatory responses involve the upregulation of IL-10 in an attempt to control the inflammation. Therefore, the level of IL-10 might be related to the prognosis of, rather than the activity of, the disease.

Finally, in our study, the expression level of IL-4 was below the detection threshold in both the peripheral blood and the AqH, which is consistent with previous studies [10], [19]. The weak correlation between VKH/BD and Th2 might be one of the mechanisms that are responsible. However, the subjects in our study were all in the inactive period, which also may have contributed to these results. In terms of IL-2, there were no significant differences among the three groups in either the AqH or the peripheral blood; this result was most likely due to the subjects being in the inactive period because previous studies have found notably increased levels of IL-2 in active VKH/BD patients.

The results and interpretation of the current study must be understood within the context of its strengths and limitations. Strengths of the study include the combination of clinical and basic research, with ideal prospective and controlled design. Translational medicine is an important direction of current medical research, which will enhance the understanding of the pathogeneses and improve the prognosis of clinical treatment of different diseases. Weaknesses of the study must also be acknowledged: though this was a controlled study, the patients could not be randomized and the number of participants is limited. Further, BD and VKH may be at different stages of disease activity and any comparison between these two diseases may be inaccurate. Last, because of ethical reason, the samples of peripheral blood and aqueous humor could only be obtained at a time point prior to their cataract surgeries and the dynamic changes of cytokines of an individual patient could not be studied in vivo. Despite these limitations, this remains one of first studies to demonstrate distinct expression profiles of cytokines and the correlations of those expression profiles with the in vivo inflammation indexes of the inflammatory and AR cataract patients. Further studies are required to identify the functions of these discrepantly-expressed cytokines (such as IL-6) and to clarify their possible roles in the formation of inflammatory cataracts.
